# Sustainability-Focused Crypto Protocols Design Methodology for Sensors and IoT with the Deployment of LLMs

**DOI:** 10.3390/s26154930

**Published:** 2026-08-04

**Authors:** Denis Trček

**Affiliations:** Faculty of Computer and Information Science, University of Ljubljana, Večna pot 113, 1000 Ljubljana, Slovenia; denis.trcek@fri.uni-lj.si

**Keywords:** sensors, internet of things, cryptographic protocols, quantum computing, authentication, key exchange, formal methods, large language models

## Abstract

Among the key computing challenges today are green computing and quantum computing, with the quantum paradigm introducing adverse consequences for many existing cryptographic protocols. However, the entire internet critically depends on these protocols. Therefore, building on theoretical preliminaries, this paper derives a coherent methodological basis for designing and developing quantum-computing-resistant and sustainable protocols for sensors and the Internet of Things (IoT) by extending traditional approaches and including large language models (LLMs). In line with these premises, this paper focuses on authenticated key exchange (AKE), which is one key security service. To support ever-growing needs under the described conditions and using the developed methodology (called the Sustainability-focused LLM-supported crypto protocols development process, SFLSCDP), this paper presents a novel, formally verified AKE protocol family. This family of protocols can be considered ultra-lightweight, as a corresponding metric is also given. Consequently, additional effective means for security services in the post-quantum computing era are provided, even for computationally weak devices.

## 1. Introduction

Many Internet services today rely on authentication, one of the key security services. For a long time, authentication was implemented with passwords, but these alone are vulnerable to many attacks. Therefore, two-factor authentication methods have been introduced, in which passwords still play an important role. These approaches are now finally being replaced by the industry-standard Passkey that excludes passwords and is being developed by FIDO Alliance [1].

Another very important cryptographic service is confidentiality, which is usually realized through authenticated key exchange/establishment (AKE for short). AKE typically takes place during the authentication phase, when the necessary material is exchanged so that the entities can derive a session key. This key is then used for symmetric encryption of bulk data during a session and is destroyed afterward. A typical example of such an approach is the Transport Layer Security protocol [2], the de jure and de facto standard for most current web transactions.

When focusing on computationally weak devices, such as the Internet of Things (IoT), the approaches described above are often not suitable or directly applicable. They are intended for traditional computers, such as desktops, laptops, and servers, and they often require biometric support. On the other hand, IoT devices frequently have limited resources, i.e., they lack computing and energy resources for traditional security provisioning. In addition, sustainability issues are becoming important. Since these devices are often powered by batteries, they require active intervention for their replacements, while the batteries themselves pose an environmental threat [3,4].

However, the number of IoT devices is increasing significantly. In 2023, we already reached 15.9 billion IoT devices, and by 2030, this number is expected to increase to 32.1 billion [5]. The security of IoT devices is therefore coming to the fore. Consequently, lightweight solutions for them are essential. Further, if such a solution is to be sustainable, it must not only operate on a low budget, but must also be resistant to emerging threats, which include the quantum computing paradigm. In addition, computing is increasingly contributing to global electricity consumption nowadays (estimates go up to 10%), at the same time producing a notable carbon footprint (about 4%, which is comparable to the aviation sector) [6]. Therefore, it is not only computationally weak devices that can benefit from solutions like the one presented in this paper.

Last but not least, another important perspective that has not received extensive attention so far is the process of designing crypto protocols. It turns out that LLMs can play a very valuable role here.

In summary, this paper focuses on the following research questions:

- Can existing lightweight authenticated key exchange (AKE) protocols for IoT and sensor networks be improved through the design of a novel lightweight protocol that also provides resistance to quantum computing attacks?
- Can a structured procedural approach to cryptographic protocol development be derived from existing design practices, and how can the integration of LLMs improve this development process to also address sustainability issues?
- Can a metrics-based framework be established to approximate the computational efficiency of lightweight cryptographic protocols while supporting future extensions for improved measurement accuracy?

In line with these research questions, the article presents novel solutions to the above problems as follows. Section 2 first introduces the theoretical preliminaries, covering the Sustainability-focused LLM-supported crypto protocols development process (SFLSCDP). This is followed by protocol evaluation, i.e., protocol metrics for assessing energy-related performance. The section concludes with theoretical preliminaries on quantum computing in relation to the post-quantum security of cryptographic protocols. Section 3 introduces a new protocol, called Ultralight Authenticated Key Exchange (UL-AKE), which is based on strong one-way hash functions and enables authenticated key exchange. Variants of this protocol are presented in the same section. The two basic versions are improved using SFLSCDP and are described in the following subsection. Formal verification issues are addressed in the final subsection. Section 4 provides a detailed quantitative evaluation of UL-AKE variant 1, compared with competing solutions. Section 5 contains a discussion and outlines future work, while Section 6 presents the conclusion and is followed by Appendix A. The paper ends with the necessary declarations and references.

## 2. Theoretical Preliminaries

Theoretical preliminaries will serve to focus on a unified development framework, which addresses security properties and efficient implementations of crypto protocols. Also, quantum computing is addressed in relation to cryptographic strong one-way hash functions.

### 2.1. Sustainability-Focused LLM-Supported Crypto Protocols Development Process

The development of a protocol is typically an iterative process, in particular its verification and optimization phases. After having the initial protocol design, the solution is first verified with an appropriate formal method, which typically belongs to the proof theory arsenal (like, e.g., BAN logic [7] for belief states verification in a system) or model theory arsenal (like AVISPA [8] for Dolev–Yao channel [9]), then optimized, verified again, etc.

**Figure 1:**
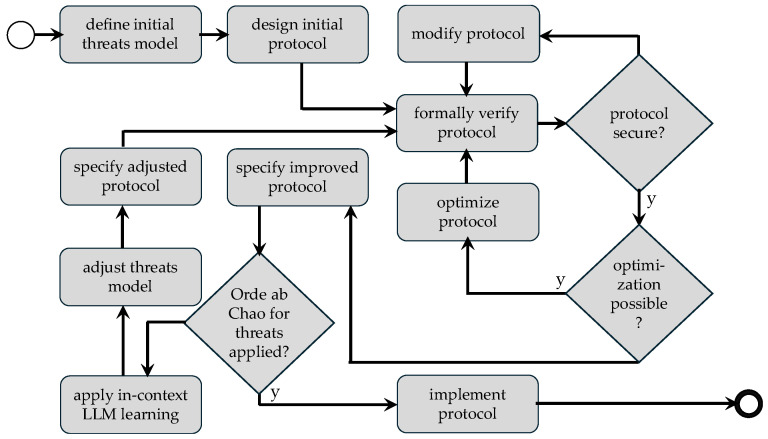
SFLSCDP methodology—a structured approach to measurable, lightweight and sustainable cryptographic protocols (two decision points in the lower part are LLM-supported).

However, approaches here vary, particularly regarding the quantitative evaluation of solutions (not to mention evolutionary perspectives via proactive threat models). If sustainability is to be ensured, the protocol must be lightweight and resistant to emerging (even yet unseen) threats. This is where the new methodology, called the Sustainability-focused LLM-Supported crypto protocols development process (SFLSCDP), comes in. It proceeds as follows (see Figure 1):

1. Define an initial threats model, which a new protocol will have to withstand.
2. Design the initial protocol that counters this initial threats model.
3. Formally verify the obtained protocol using a suitable formal method that corresponds to the current threats model. Make the necessary security modifications, if needed.
4. If optimization is possible (including involvement of LLMs), optimize the protocol and go to step 3; else go to the next step.
5. Use procedural prompting for an in-context learning of the selected LLM so that the model can deploy an appropriate method—in the case of threats, such a method (which is based on creative, so-called lateral thinking [10]) is the Orde ab Chao method [11].
6. Present the in-context learnt LLM with your threat model and let the LLM expand it further by incorporating evolutionary views (e.g., quantum computing).
7. Evaluate the newly obtained threat model. If threats exceed the nature of the initial one, identify an appropriate formal method. If such a method exists and has not yet been deployed, go to step three; else go to the next step.
8. If the LLM did not extend (the nature of) the initial threats, implement the protocol with clearly stated limitations in line with the initial threats model. Else, implement the protocol in line with the adjusted threat model, verify it formally, and clearly state the corresponding threat model limitations as well. But if formal verification is not yet possible, state this clearly as well, together with eventual implementation.

Emerging threats require further elaboration, as this issue relates to certain forecasting capabilities. Lateral thinking techniques invented by De Bono can be successfully deployed for this purpose. However, in their basic form they do not address so-called disruptive developments. Security threats are often of this nature, so an extended lateral thinking-based method called Orde ab Chao was developed. Although Orde ab Chao is intended for humans, recent groundbreaking developments in LLMs enable its further applications. Instead of humans, procedural prompting for in-context learning of LLMs can be used for identification of disruptive threats (described in the next section).

### 2.2. Quantitative Performance Metrics for Protocols

Research on lightweight protocols inevitably involves metrics to quantify how lightweight a given solution is. However, the actual energy a protocol requires is only known after its in silico implementation. Evaluating its software implementation is often inappropriate, as IoT devices may be fully implemented in hardware or may use non-traditional architecture. Thus, a more abstract, yet still sufficiently precise, power consumption model can be employed. Two main approaches exist, which are analyzed below:

- The first approach is based on the equivalent number of gates or GE (see such evaluations in, e.g., [12,13], where the corresponding theoretical basis can be found in [14]). Since the number of gates is related to power consumption, GE is the basis for the power consumption of a protocol. More precisely, each CMOS gate (CMOS is the predominant technology today) consists of transistors whose power consumption is given by the following equation [15]:
  $${P≅P_{s}+P_{d}=(I}_{l}V_{DD})+({fC}_{l}V_{DD}^{2}).$$

In the above equation P is the total power consumption, consisting of static consumption $P_{s}$ and dynamic consumption $P_{d}$ (the so-called direct-path current-related power is neglected). The first summand is due to the leakage of a transistor, while the second one is due to the switching ($V_{DD}$ stands for supply voltage, $I_{l}$ for leakage current, f for switching frequency and $C_{l}$ for load capacitance, which is proportional to the physical dimensions of the transistor). The main power consumption comes from switching load capacitance.

- The second approach is based on idealized times for a given crypto operation (see such evaluations in, e.g., [16,17], while an improved theoretical basis can be found in [18]). To determine energy consumption, the maximum performance capacity of the CPU is taken and multiplied by the total time for the execution of cryptographic operations and communication. More precisely, the idealized times mentioned are given for typical operations such as hashing, i.e., *T*_H_, or symmetric encryption, i.e., *T*_S_. These times are summed up to obtain the estimated total time in its idealized form, which serves as a basis for comparing solutions. In addition, the consumption of the transmitted messages is measured in bits, which is determined by simulations. Usually, a protocol is implemented using a crypto library (such as PyCrypto) and the implementation of the protocol is measured in a simulator such as NS-2/3.

The first type will be referred to as direct power/energy consumption (DPEC), while the second type of measurement will be referred to as indirect power/energy consumption (IPEC). Although both types are approximate, they require further elaboration to obtain more realistic figures.

With IPEC, the description of the measurement environment is often as follows: “The system is configured with Ubuntu 19.04, 16 GB RAM and a 3.60 GHz Core i7 processor to run the authentication techniques…” [16]. Such an inadequate description leads to overly rough estimates of consumption and problems with general comparability. For example, when providing simulation results, the exact CPU time for a protocol remains problematic, as it can be influenced by system characteristics such as bus and RAM bandwidth and latency, cache size and speed, hardware acceleration features, and concurrency with multithreading. The way the operating system provides access to the CPU time allocated for protocol execution also plays a role. Furthermore, many protocols do not run on von Neumann architecture, etc. Therefore, for broader comparability, the metrics rely on idealized times for local computations, while communication costs are still expressed in bits, as further refinements would introduce large deviations.

### 2.3. Quantum Computing Threats

The advances in quantum computing are already showing promising results. Although quantum computing is not a panacea for all problems, for certain problems (like factoring large prime composites), it is [19]. Currently, quantum computers can effectively deploy some tens to hundreds of qubits [20], but due to notable developments, this situation must be considered seriously. In the case of strong one-way hash functions, Grover’s algorithm plays a crucial role [21], and it will be summarized next (based also on the work described in [22,23]).

**Figure 2:**
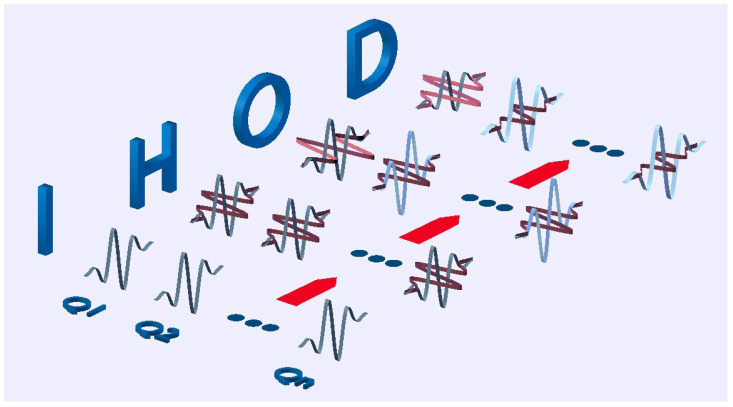
Idealized Grover’s algorithm where *I* stands for initialization, *H* for Hadamard operation, *O* for oracle application and *D* for diffusion operator (brighter packets represent correct values, while red arrows indicate the sequence of applied operations).

Informally, Grover’s algorithm goes as follows. Initially, all the qubits are put into the state ∣0⟩ (zero state). Next, all the qubits are subject to a Hadamard gate that puts all qubits into a superposition. Afterwards, the quantum oracle is applied, which marks the appropriate solution by reversing its phase (the correct solution means the sequence of bits in these qubits). Next, the diffusion step follows, which averages and reflects around the average the marked solution. Consequently, the amplitudes of correct values are enlarged and reversed in phase, while other amplitudes are lowered. The oracle and diffusion steps are repeated an appropriate number of times to reach the optimal state for measurement, where the probability of a correct result is highest. The whole procedure is conceptually presented in Figure 2 (note that this is an idealized representation, where qubits Q_1_ to Q_*n*_ are represented as wave packets and superpositions of qubits are denoted as two orthogonal wave packets).

Now, formally, suppose we have a function f:{0,1,…,N−1}→{0,1}, whose domain represents indices to database entries. This database is unstructured and $N=2^{n}$, where *n* is the number of qubits. The function *f* maps an index to 1 iff f(x)=1, otherwise f(x)=0 (meaning in general that x=ω, where ω is the index of an entry that matches the search criterion).

Qubits in state 0 are denoted by ket $|0\rangle =\;\left[\begin{array}{c} 1\\ 0 \end{array}\right]$, and when in state 1, they are denoted by ket $|1\rangle =\left[\begin{array}{c} 0\\ 1 \end{array}\right]$ (note that bra notations of these two state vectors are $\langle 0|=\;\left[\begin{array}{c} 1\;0 \end{array}\right]$ and $\langle 1|=\;\left[\begin{array}{c} 0\;1 \end{array}\right]$, respectively). When in superposition, the qubit state is given by the equation ∣ψ⟩=α∣0⟩+β∣1⟩, where ψ is a new state vector of the qubit, while α and β are complex factors, referred to as probability amplitudes. When measured (measurement causes the new state vector to collapse), the squares of probability amplitudes give the probability of a particular bit value as an outcome. Therefore, ${|\alpha |}^{2}+{|\beta |}^{2}=1$.

Before putting qubits into superposition, one first initializes *n* qubits to the zero state, where $n={log}_{2}\left(N\right)$, while *N* is the number of items in an unsorted list. These qubits are initialized as follows:

(1) $${|0\rangle }^{⊗n}=|0\rangle |0\rangle \cdots |0\rangle =|0\;0\ldots 0\rangle .$$

Next, the Hadamard gate operation H is applied to each qubit, which in linear algebra is represented by the following matrix:

(2) $$H=\left[\begin{array}{cc} \frac{1}{\sqrt{2}} & \frac{1}{\sqrt{2}}\\ \frac{1}{\sqrt{2}} & -\frac{1}{\sqrt{2}} \end{array}\right]=\frac{1}{\sqrt{2}}\left[\begin{array}{cc} 1 & 1\\ 1 & -1 \end{array}\right].$$

After the application of *H* gate, the system is in a new superposition state ∣s⟩:

(3) ∣s⟩=H⊗n∣0⟩⊗n=12n∑n=0N−1∣x⟩.

Next, an oracle *O* is used. An oracle is an application for determining a certain property of a function, and quantum computers can determine this property globally on a selected function, not just for a particular input (i.e., particular argument of the function). The oracle operation is as follows:

(4) $$U_{\omega }|x\rangle =\{\begin{array}{c} -|x\rangle \;\;\;\;\;\mathrm{if}\;x\;=\omega ,\;\mathrm{that}\;\mathrm{is},\;f(x)=1\\ \;\;\;\;|x\rangle \;\;\;\;\;\mathrm{if}\;x\;\neq \omega ,\;\mathrm{that}\;\mathrm{is},\;f(x)=0 \end{array}\;.$$

This operation tags the correct solution by flipping the sign of the probability amplitude of the target state. Next comes the application of the diffusion operator $U_{s}$, which operates on the obtained superposition state as follows (I denotes the identity matrix):

(5) $$U_{s}=\left(2\left|s\right\rangle \;\left\langle s\right|-I\right).$$

Using Equations (4) and (5), one complete iteration of Grover’s algorithm is performed:

(6) $$G=U_{s}\cdot U_{\omega }.$$

Note that the above operation is run on n qubits, in parallel. The optimal number of iterations (which maximizes the chance of obtaining the correct result) is approximately $r\approx \frac{\pi }{4}\sqrt{N}$ [23].

Grover’s algorithm reduces the asymptotic complexity of finding the correct entry in an unsorted database from O(N) with a classical computer to *O*($\sqrt{N}$) with a quantum computer, providing a quadratic speedup. In other words, the time required by the quantum computer to solve the search problem is the square root of the time required by a classical computer. This is important for attacks on one-way hash functions, especially on their pre-image resistance (it is easy to compute the output from an input, but given only the output, finding its input, i.e., the pre-image, is hard).

Although this mapping is deterministic, it generates the outputs in such a way that ideally, they are randomly and uniformly distributed over the codomain. Due to the aforementioned properties, an attacker must perform a brute-force attack to find the pre-image of a specific output, which has a complexity of $O\left(2^{n}\right).$ As Grover’s algorithm provides quadratic reduction, the complexity is $O\left(2^{n/2}\right)$.

Resilience to quantum computing can therefore be achieved by using hash functions that provide twice the number of output bits. But hash functions are also vulnerable to attacks, where two different inputs are mapped to the same output and this output is not fixed—it can be an arbitrary one. The attacks that exploit this property are referred to as collision attacks, which have the complexity $O\left(2^{n/2}\right)$ [24]. As such attacks are inherent in hash functions, today their outputs’ length is often adjusted accordingly, i.e., doubled.

## 3. Novel Protocol Derivation

In this section, we first present a novel protocol for ultra-lightweight authenticated key exchange (and subsequent provision of confidentiality), called UL-AKE. It uses only strong one-way hash functions, along with concatenation and XOR operations. Next, in line with our methodology, we apply an LLM to identify possible improvements to the protocol.

### 3.1. Basic UL-AKE Protocol Derivation

The core pieces of UL-AKE are strong one-way hash functions to achieve effective, non-burdensome computations (as opposed to public-key cryptography, for example). To further improve their effectiveness, having only one cycle of a hash function is preferred. Thus, in the case of SHA-256 [25], UL-AKE focuses on ensuring 447-bit-long inputs (longer inputs require additional cycles). Recall that one cycle of SHA functions involves initializing registers A, B, C, D, E and then processing that state in 80 steps, with four sets of different primitive functions, additive constants, derived words, applying also circular register shifts, etc. (SHA-3 processing is a bit different [26], but the reasoning remains the same). This is more costly than performing XOR operations on the arguments first and then hashing the result. However, XOR operations can (and most often must) be replaced by concatenation to provide higher security—this will be elaborated in more detail below.

There are two variants of UL-AKE. The first one, UL-AKE variant 1, is computationally more efficient but less secure, and should be used with caution. All hash arguments are fixed-length bit strings, and for today’s purposes these lengths are sufficient: principals’ IDs are 128 bits long, random values are 256 bits long, and the secret symmetric key is 128 bits long. When the concatenation of these arguments exceeds 447 bits, they are XORed, and the result is hashed afterward.

The approach described may not always be acceptable; therefore, the second variant, UL-AKE variant 2, is used. With this variant, the input arguments are simply concatenated and hashed with SHA-256 like any other bit string. Consequently, more iterations of the compression function may be performed, resulting in a more secure output.

To further reduce costs, many competing protocols, analyzed in Section 4, use timestamps and then compute time differences to avoid indefinite waiting for the next message (if a previous one has been lost or tampered with). This requires a synchronized time base, which is more costly than a local timer implementation with random values, as in the case of the UL-AKE family. For the storage of random values, each device stores and compares them with newly received ones to check message freshness (i.e., whether it belongs to a particular run of a protocol). Since IoT devices have limited resources, a low-capacity FIFO memory is used. When a new message arrives, its value is stored and the oldest one is discarded.

**Figure 3:**
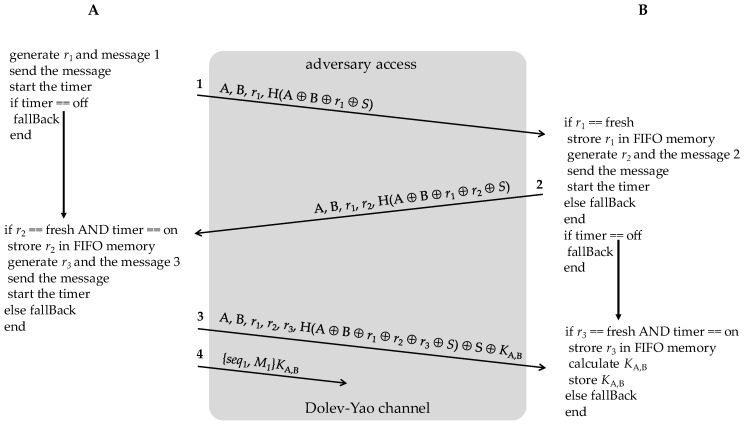
Authentication and key-exchange phases of UL-AKE variant 1 protocol (the general checks of received messages are described in the text).

The complete UL-AKE protocol variant 1 is now given in Figure 3. The meaning of the symbols is as follows: A and B denote principals’ IDs (among which there is at least one IoT device), *r*_1_, *r*_2_ and *r*_3_ are random values, *S* is a shared secret between A and B, *K*_A,B_ is a secret shared symmetric key between A and B, while *M* denotes the message to be encrypted.

Now A generates *r*_1_ and creates the first message. It sends it to entity B and starts its timer. The timer prevents A from waiting indefinitely for a response if, for example, the message is lost. After receiving the message, entity B first carries out a general check of the message. This check includes the structure of the message for a specific step. If the check fails, B discards the message and returns to the initial state to wait for a correct message. If the general check is passed, entity B starts the security-related check of the message by using the arguments received in plain text, verifying them (the identifiers and the uniqueness, i.e., the freshness of the random values), XORing them, hashing them and comparing the result with the received hash value. If the check is successful, B stores the random values for future freshness checks of the received messages. Next, it generates *r*_2_, uses the remaining (stored) arguments, XORs and hashes them to obtain message 2. Now B starts the timer and sends the message to entity A.

If entity A has not yet returned to the initial state after receiving the second message due to the timer expiring, it performs the same procedure as described above for B, which is extended by the inclusion of *r*_3_ and *K*_A,B_. Afterwards, it sends message 3 to B, followed by the confidential payload *M*_1_ from A in message 4. It starts the timer and enters the state in which it expects an encrypted payload response from B. After the first three steps, A and B are thus mutually authenticated and share a secret session key *K*_A,B_. Now the confidential transmission of bulk data (i.e., the messages *M*_1_, *M*_2_, …) can take place. The messages must use consecutive numbering to prevent reply attacks.

Clearly, this variant of UL-AKE, where arguments are XORed before hashing, increases opportunities for attackers, as different operands can produce the same output and thus present a possible attack path (recall that XOR is commutative, associative, and not injective). Plain concatenation of arguments provides higher long-term security. So, if the concatenation of the arguments results in bit length larger than one input block, besides UL-AKE variant 2, a different hash function can also be used with a larger input size like the SHAKE family function [26].

Final notes related to UL-AKE variants’ security. In line with the selected model, the requirements for the protocols are as follows: B authenticates A on *r*_1_, A authenticates B on *r*_1_, and B authenticates A on *r*_3_. The following assumptions are therefore proved by AVISPA: secrecy (i.e., confidentiality) of *K*_A,B_, *M* and *S*. The hash function is treated as an idealized primitive, which serves as a MAC. Inclusion of *r*_1_, *r*_2_ and *r*_3_ in messages 1, 2, and 3 ensures freshness of the exchanged messages (to prevent reply attacks). Their inclusion (their sequence) in hashing ensures the integrity of the whole protocol run. However, no security is claimed under the random oracle model—the only additional claim is the one given about quantum computing resistance (see details in Section 3.3).

### 3.2. UL-AKE Protocol Improvements—UL-AKE-KD

In line with SFLSCDP, the LLM inclusion step follows, and it should be emphasized that a locally run LLM was used. Using procedural prompting, the LLM first learned the core in-context part, i.e., the description of UL-AKE variant 1. Next, the model was instructed to find possible optimizations and improvements.

For variant 1, the LLM first identified the previously mentioned issues related to XORed hash arguments (i.e., it suggested using concatenation for hashing the arguments). It also suggested using more recent principles, where the session key is not sent over the network but generated on each side independently using a key-derivation (KD) step. The suggested UL-AKE-KD variant steps go as follows (concatenation is denoted as “∥”):

1. $A\to B:\;A,B,r_{1},H(A∥B∥r_{1}∥S)$;
2. $B\to A:\;A,B,r_{1},r_{2},H(A∥B∥r_{1}∥r_{2}∥S)$;
3. $key\;derivation:\;K_{A,B}=H(S∥r_{1}∥r_{2}∥r_{3})$;
4. $A\to B:\;A,B,r_{1},r_{2},r_{3},H(K_{A,B}∥r_{3})$;
5. $A\to B:\;\{{\mathrm{seq}}_{1},M_{1}\}K_{A,B}$.

Next, the in-context learning with the Orde ab Chao method for disruptive innovations was performed. Due to the limited LLM context size, only a minimal version (compared to that mentioned in [27]) was used. LLM was instructed to find new, disruptive threats that fall out of the scope of the Dolev–Yao model (explained in detail in Section 5).

The initially identified threats included side-channel attacks, implementation vulnerabilities, hardware-focused attacks, and generic crypto-mechanisms (hash functions in our case) attacks like length extension attacks. After being instructed on disruptive threats, the model identified additional, already expected disruptive developments. These included quantum computing, AI-driven exploits, edge and fog vulnerabilities, open-source hardware, 6G with massive IoT, and zero trust architectures.

### 3.3. Formal Verification of UL-AKE

UL-AKE, both variants, and UL-AKE-KD have been formally verified with AVISPA v 1.1, one of the most successful security verification tools with an extensive track record. AVISPA assumes communication over the Dolev–Yao channel, where abstract machines exchange messages, and these messages consist of formal terms. Part of the internal structure of the messages can be disclosed, while some parts must remain inaccessible to an adversary. Furthermore, according to AVISPA’s assumptions, the hash function is treated as a public strong one-way function. 

The protocols were specified in a user-friendly High-Level Protocol Specification Language (HLPSL), which is subsequently converted into a less readable Intermediate Format (IF). IF then drives four backend test programs: the on-the-fly model-checking program OFMC, the constraint logic-based attack search program ATSE, the SAT-based (Boolean satisfiability problem) model-checking program SATMC, and the automatic approximation-based tree automata program for security protocol analysis (TA4SP).

UL-AKE variant 1 and variant 2 were verified with the OFMC and ATSE backends (the SATMC and TA4SP backends do not support the XOR function). Both marked the protocols as “SAFE.” UL-AKE-KD was also checked with OFMC and ATSE, and additionally with SATMC/SIM; all reported the protocol as “SAFE,” indicating that no attacks were found within their respective analysis models. Only the TA4SP backend returned “INCONCLUSIVE.” This should not be interpreted as evidence of a protocol weakness; rather, it reflects limitations of the analysis technique, which may be unable to determine whether the protocol is secure (the process ended with a timeout).

The HLPSL specifications for all the protocols can be found in [28].

## 4. Quantitative Evaluations

This section presents the first quantitative evaluation of UL-AKE variant 1, the lighter of the two, followed by evaluations of its competitors. To improve readability, IPEC was used for the first round of evaluation. Afterwards, the two most promising candidates, together with UL-AKE variant 1, are evaluated further with DPEC.

### 4.1. UL-AKE Variant 1 Evaluation

UL-AKE variant 1 will be compared to the protocols analyzed in the next subsection using GE. The GE metric is based on the area corresponding to a two-input NAND gate, because these gates not only allow the implementation of any Boolean function but are also the most technologically and economically advantageous. GE figures for key structures for (asynchronous) RFIDs are as follows: The number of transistors for a 2-input NAND gate is 4, for the 2-bit input XOR operation GE is 4 [15], and for storage (D latch) is 5 NAND gates [13]. For the pseudo-random number generator (where a shift register with linear feedback, LFSR, is typically used), *n* latches and one XOR gate are required for an *n*-bit-long output [29]. For AES-128, the GE is 2400 [30], while for SHA-256 it is ~8600 [31]. Next, an astable multivibrator can be used for a timer, which requires 2 NAND gates [32]. Finally, to put things in perspective, the GE value for elliptic curve-based cryptography is 25,000 [33], while some less common, but less scrutinized public key cryptosystems can have a much better GE number, such as 4682 [34].

Analyzing now UL-AKE variant 1 with IPEC metrics—the whole AKE phase requires three hashing operations, i.e., 3 *T_H_*, and 14 XOR operations, i.e., 14 *T_XOR_*. However, one should consider each entity, so entity A needs 2 *T_H_* and 10 *T_XOR_*, while entity B (typically an IoT device) needs only 1 *T_H_* and 4 *T_XOR_*. Furthermore, all protocols analyzed in the next subsection work with concatenated arguments for hash operations. UL-AKE variant 1 avoids concatenation of arguments, as this can lead to additional cycles when hashing, which is more expensive than a few additional XOR operations. But UL-AKE variant 2 does operate on concatenation of arguments to increase security.

Now the resources for UL-AKE variant 1 are as follows (if three XOR operations are required, the cost is calculated as twice GE for performing one XOR operation; freshness checks are excluded, the cost of message formation is also excluded, while the timer costs can be neglected):

1. In step 1, for storing identifiers A and B, 128 bits are needed for each (for compliance with IPv6 addresses). This requires 2 × 5 × 128 NAND gates. Random value *r*_1_ is 256 bits long, so approx. 5 × 256 NAND gates are needed for its generation with LSFR. Secret value *S* is 256 bits long, requiring 5 × 256 NAND gates for its storage. XOR operation is performed three times on 256-bit values, thus requiring 2 × 4 × 256 NAND gates; subsequently, this value is hashed, requiring 8600 NAND gates and stored again, which is 5 × 256 NAND gates—total approx. 2 × 5 × 128 + 5 × 256 + 5 × 256 + 2 × 4 × 256 + 8600 + 5 × 256 = 15,768 NAND gates.
2. In step 2, in addition to the resources required in step 1, *r*_2_ is generated and stored, requiring an additional 5 × 256 NAND gates for generation and 5 × 256 NAND gates for storage. An additional XOR operation is needed, requiring 4 × 256 NAND gates, and hashing needs 8600 gates, while storing the result requires 5 × 256 gates—total approx. 15,768 + 5 × 256 + 5 × 256 + 4 × 256 + 8600 + 5 × 256 = 29,232 NAND gates.
3. In step 3, the additional cost added to the cost in step 2 is another random value generation with storage and session key generation with storage (128 bits, as AES is assumed for subsequent communications). Further, additional gates for three XOR operations are needed, while hashing needs 8600 gates, and output’s storage needs 5 × 256 gates—total 29,232 + 5 × 256 + 5 × 256 + 5 × 256 + 5 × 256 + 5 × 128 + 5 × 128 + 2 × 4 × 256 + 8600 + 5 × 256 = 47,560 NAND gates.

The total number (without communication cost) is approx. 15,768 + 29,232 + 47,560 = 92,560 NAND gates. It should be noted that this refers to the total energy consumption for the entire protocol, of which ~60,000 NAND gates are required for one device and ~30,000 for the other. For example, the solution given in [12] requires 7 × 3520 × 5 = 123,200 NAND gates only for the exchanged messages (this protocol supposedly has better performance than the seven others analyzed in [12]). In the case of UL-AKE v1, the length of the exchanged messages is 2 × 128 bits for IDs, while *r* and *S* require 256 bits each. Thus, the length of message 1 is 2 × 128b + 256b + 256b = 768b, the length of message 2 is 2 × 128b + 2 × 256b + 256b = 1024b and the length of message 3 is 2 × 128b + 3 × 256b + 256b + 256b + 256b = 1792b. The total number of bits is 768b + 1024b + 1792b = 3584b. The GE for the communication costs is 5 × 3584 NAND gates = 17,920 NAND gates. The overall GE of UL-AKE variant 1 is 92,560 NAND gates + 17,920 NAND gates = 110,480 NAND gates.

### 4.2. UL-AKE Competition Evaluation

One extensive recent survey on AKE can be found in [16]. Of the numerous protocols mentioned there, only those that provide mutual AKE for subsequent confidentiality are chosen. This results in a selection of fifteen protocols, which is further narrowed down to those that do not use asymmetric encryption, but are based on cryptographic hash functions and XOR operations. The reason for this is not only the desire for lightweight implementations, but also the focus on resistance to quantum computing. The remaining six protocols are then complemented by the recent research on lightweight protocols given in [12,17]. The first reference provides additional recent publications besides the above-mentioned studies, while the second one deals with Radio Frequency Identification Tags (RFIDs), which are the weakest representatives of the IoT world (it also provides a good overview of other lightweight RFID protocols).

The eight protocols obtained are analyzed in more detail next, with the focus on the AKE phase. Starting with a lightweight scheme targeting wireless sensor networks (WSNs), presented in [35] and enumerated as I in Table 1—it is based on hashing and XOR operations, concatenation operations, generation of random/time values and their processing (comparisons). Its AKE consists of 5 steps and 4 exchanged messages. The messages are relatively complex to process. For example, message 1 requires 5 hashing operations (on concatenated arguments) and 4 XOR operations. The protocol was formally verified using the Proverif tool [36]. Idealized operation times are given for the analysis of the computing power, while the communication costs are 2720 bits, which were determined by a simulation with NS-3 on Ubuntu 16.10.

In [37] (enumerated as II in Table 1), another efficient AKE for WSNs is presented. The scheme for multi-gateway-based WSNs uses hash functions and XOR operations (besides concatenation and random or timed value generation, verification and storage, which are not further mentioned here). There are two options for AKE. The shorter option has 4 steps with 3 exchanged messages. The most complex messages are the first and third messages, with the first message alone requiring 5 hashing operations (on concatenated arguments) and 4 XOR operations. The security is formally checked with Proverif. For performance metrics, the idealized times for hash operations are given, while XOR operations are not considered. Furthermore, the communication cost in bits is 2688 for the first, less demanding option. These costs are evaluated in terms of the time required (to consequently determine power consumption) and by using the NS-2 simulator.

The protocol for body area sensor networks (BANs) is presented in [38] (enumerated as III in Table 1). It provides mutual authentication and key exchange between a user (i.e., her IoT devices) and a personal server connected to the WBAN via a healthcare server. The protocol uses hash and XOR operations. In addition, it uses biometrics and consequently fuzzy extractors. Its AKE consists of 4 steps with 3 exchanged messages, with the processing of the second message (the most processed) requiring 15 hash operations and 5 XOR operations. In terms of security, the protocol is informally verified using the Real-Or-Random model, while performance analysis is performed using the NS-2 simulator on Ubuntu 14.04 LTS. The communication cost for each of the three required messages is 1696 bits, and this cost is then converted into time values using the simulation.

The solution presented in [39] (enumerated as IV in Table 1) provides authentication and key exchange (as well as the ability to change passwords) for vehicle sensor networks (VSNs). It uses XOR and hash operations. The AKE part consists of 5 steps with 4 exchanged messages. The most extensive message requires 5 hash operations and 5 XOR operations (again, as with the above protocols, hashing is performed on concatenated arguments). The protocol is informally verified for its security properties. In addition, its overall performance, which is evaluated using idealized times, requires 20 *T*_H_ (and a total of 16 XOR operations) together with 1280 bits for communication.

In [40], edge computing is discussed, which includes IoT devices, edge nodes and cloud servers (enumerated as V in Table 1). The IoT device authenticates itself to a cloud server supported by an edge node. If successful, a session key is established between the two. Formal verification is done using AVISPA [8], while performance is estimated using NS-2 simulation. Analyzing AKE and focusing only on the processing of the last message (performed by an IoT), this requires 10 hash operations on concatenated arguments and 4 XOR operations on arguments with hash output length. The remaining four steps (and the resulting messages) are of comparable complexity for processing.

The solution in [41] (enumerated as VI in Table 1) supports IoT devices for connections with other IoT devices or a server via AKE and also provides anonymity. It comprises only two units: an IoT device and a cloud server. The number of messages exchanged is low (only three in the AKE phase). However, it relies on public-key cryptography (elliptic-curve-based cryptography), accompanied by hash operations on chained arguments, plus some XOR operations. The solution has been formally verified with AVISPA, while the performance analysis is based on memory consumption and computational costs in terms of idealized execution times. For communication, the cost is 1408 bits. Although the solution uses public-key cryptography, it is included as it is very minimal in other respects.

A recent lightweight protocol from [17] should also be mentioned (enumerated as VII in Table 1), which has been formally verified with Proverif, assuming the Dolev–Yao model. It is intended for smart grids to provide AKE between smart meters and power suppliers’ servers via neighborhood gateways. It is based on hash functions and XOR operations. It consists of initialization and two additional phases with few exchanged messages. In the first phase, it uses 1 hash and 1 XOR operation on the smart meter side, while in the second phase the smart meter has to perform an additional 4 hash operations (with two different hash functions) and two additional XOR operations—a total of 5 hash operations (on concatenated arguments) and 3 XOR operations. Its overall performance (besides the AKE phase) requires 10 *T*_H_ (hash operations). For the GE metric and the first phase of the protocols, the computation goes as follows:

1. Generation of a random value *r* and its storage at the gateway side.
2. Storage of a secret message *m* and a symmetric key *K*.
3. Computation of A=((m ⨁ r)∥r) ⨁ K and V=H(m∥r∥ID∥T∥K), and storage of *A* and *V*, where *r* denotes a random value, *T* denotes a timestamp and *ID* is the identifier of a smart meter.
4. Sending *A*, *V*, ID and *T*.
5. Storing of the received *A*, *V*, ID, *T* values at the meter side, where also the secret key *K* is already stored.
6. Computation of ((m ⨁ r))∥r) to obtain A ⨁ K.
7. Verification of *V* by computing H(m∥r∥ID∥T∥K).

To obtain the final GE estimate, some assumptions are needed (these are aligned with corresponding values used for UL-AKE evaluation). All values are represented as plain binary values; principals’ IDs are 128 bits long, random values and timestamps are 256 bits long, while the secret symmetric key is 128 bits long. The hash function used is SHA-256, so the secret message *m* is up to 447 bits long (these assumptions are the same as for the UL-AKE family). Consequently, step one needs 256 × 5 + 4 = 1284 NAND gates (*r* generation) plus 1280 NAND gates (for storage of *r*), so the total is 2564 gates. Next, step 2 requires storage for *m* and *K*, which is (512 + 128) × 5 = 3200 gates. Next, step 3 needs 2048 gates (for the XOR operation), and to perform concatenation with *r* it needs an additional 1280 storage gates, so a sub-total for *A* is 3328 gates. Adding now 2048 gates for the XOR operation with *K*, the total for *A* is 5376 gates. For *V*, the storage cost is (512 + 256 +128 + 256 + 128) × 5 = 6400 gates, but as the argument for hashing is 1280 bits long, hashing requires 3 rounds of SHA-256 application (up to 447 bits requires one compression run). So, the cost for *V* is 3 × 8600 + 6400 = 32,200 gates, and the total for step 3 is 37,576 gates. Similarly, step 4 requires (5376 + 32,200 + 128 + 256) × 5 = 189,800 gates, step 5 requires 189,800 + 128 × 5 = 190,440 gates, step 6 requires 3328 gates, and step 7 needs 32,200 gates. The grand total for authentication phase 1 in GE is 2564 + 3200 + 37,576 + 189,800 + 190,440 + 3328 + 32,200 = 459,108 gates.

And the last competitor—a special type of protocol—is those for RFIDs, which consist of several thousand logic gates. In addition, these devices are usually passively powered, which significantly limits their energy resources. Therefore, their AKE protocols must be lightweight. A good overview of such protocols with a new one can be found in [12] (enumerated as VIII in Table 1). This solution provides authentication using strong one-way hash functions and XOR operations. It has eight steps, with two steps for the RFID tag, which require 4 hash and 5 XOR operations. The protocol has been formally verified with AVISPA, Scyther [42], and manually with GNY logic [43]. As to GE, the assumptions as stated earlier must be considered. In addition to random values, the protocol uses externally synced timers, and to further preserve the readability of the text, timestamps and related processing will be omitted. Even with these assumptions, and by analyzing only the processing cost at the tag side and adding all exchanged messages, the GE is comparable to that of UL-AKE variant 1:

- In step 4, the tag generates *r*_1_ and uses a secret *S* to compute these values: $C=H(ID\;∥\;S)\;⨁\;r_{1}$, and $D=H\left(\left((ID∥S)⊕r_{1}\right)∥r_{2}∥r_{3}\right)$. (This is a possible correction of message *D*, because its structure is undefined in the original paper—one parenthesis is missing. The issue cannot be resolved, as it is also present in the text of the referenced article).
- In step 8, it computes $H(ID\;⨁\;r_{3}\;∥\;r_{1})$. Having another such structure stored from the previous steps (overlined below), it now compares the two, i.e., $H(\bar{ID}\;⨁\;\bar{r_{3}}\;∥\;\bar{r_{1}})\;\overset{?}{=}H(ID\;⨁\;r_{3}\;∥\;r_{1})$, and updates secret values $S\leftarrow S\;⨁\;r_{2}$.
- During these 8 steps, the protocol requires seven exchanged messages.

In step 4, the computation of *C* requires (128 + 256) × 5 + 8600 = 10,520 gates for hash calculation; the XOR operation requires an additional 512 × 4 = 2048 gates, while generation of *r* with its storage requires 2564 gates—so the total for *C* is 15,132 gates. Similarly, for *D*, storing the concatenation of ID and *S* requires (128 + 256) × 5 = 1920 gates, and XOR-ing this result with *r*_1_ requires 1536 gates. Further concatenations with *r*_2_ and *r*_3_, require additional (256 + 256) × 5 = 2560 gates. Finally, hashing (384 + 512) = 896 bits requires two cycles, i.e., 8600 × 2 = 17,200 gates. The cost for *H* is therefore 1920 + 1536 + 2560 + 17,200 = 23,216 gates, and the total cost for step 4 is 23,216 + 15,132 = 38,348 gates. For step 8, its approximate GE is 256 × 4 = 1024 gates for XOR, additional (256 + 256) × 5 = 2560 gates for storage of XOR-ed value and concatenated random value, while hashing this concatenation requires two cycles, i.e., GE is 2 × 8600 = 17,200 gates—the sum is 1024 + 2560 + 17,200 = 20,784 gates. Adding storage of another result for comparisons requires an additional 512 × 5 = 2560 gates, so the approximate total for step 8 is therefore 23,344 gates. Considering now all the exchanged messages, the first one requires 1024 bits, the second one 512 bits, the third one 1280 bits, the fourth one 1536 bits, the fifth one 2560 bits, the sixth one 1536 bits and the last one 768 bits—the GE for communication is thus 46,080 gates. Finally, the total GE for step 8 is 38,348 + 23,344 + 46,080 = 107,772 gates.

The results are summarized in Table 1, and they indicate that UL-AKE variant 1 is aligned with its competition. But judging it as superior to the rest of the competition requires caution, as will be discussed in the next section.

To conclude this section, the main latest lightweight AKE solutions are added, where a brief analysis reveals that their performance does not exceed the so far (in detail) presented solutions. In [44], an IoT scheme for telemedicine is given that utilizes physically unclonable functions (PUFs) together with elliptic curve-based cryptography. It consists of several phases, where already the registration phase requires 7 hash operations and 4 XOR operations (besides the rest of the operations). Further, in [45] another IoT solution, focused on advanced metering infrastructure, is given. It also deploys Chebyshev polynomials and consists of several phases: initialization, registration, mutual authentication and session key agreement, and multicast key generation. The most demanding phase, i.e., the mutual authentication and session key agreement phase, requires 13 hash operations and 4 XOR operations (besides the rest of the operations and phases)—again exceeding the performance of the already analyzed solutions.

## 5. Discussion and Future Work

Starting now with the research questions—the answer to the first research question is positive. The answer to the second one is also positive, but some further justification is needed. Finally, the answer to the third research question is only partially satisfactory. As to additional justifications of results related to the second research question: to secure the authorship of the presented research until its publication, the LLM was deployed locally. This led to limited LLM capabilities (see the technical details about the model and its deployment environment specifications in Appendix A).

Despite this, several important issues were identified, presented in this research, and elaborated in the article (see Section 3.2). Only the attempts to obtain unforeseen threats yielded no breakthrough results. The most probable reason is the relatively low parameter count of the deployed model and the comparatively weak computational environment used for its deployment. It can be reasonably assumed that the threat model could also be improved if more powerful publicly accessible LLMs were deployed (see proof in a similar research case presented in [27]).

The limitations of using LLMs in research also include more generic factors. LLMs are inherently probabilistic, and their responses depend on numerous variables that are difficult to control. The most important ones are sampling parameters and the contents of the context window. Also, far from negligible are the effects of prompt wording, conversation history, any provided examples, and system instructions. These latter factors are beyond a researcher’s control when using the most powerful online models, as they govern general model behavior, safety, and related aspects.

The application of LLMs to security, as implemented in this paper, is rather novel and, to the best of our knowledge, is the first of its kind. But LLMs have already been included in many security areas like penetration testing [46], where LLMs excel in specific sub-tasks such as testing tools deployment, interpretation of outputs, and indication of subsequent actions. On the other hand, main difficulties here include maintaining the whole context of the testing scenario. They have also been deployed in proactive threats management, based on a prognosis of disruptive developments [27]. They clearly provide very rich and numerous outputs. However, these require deeper expert inspection, as their own interpretation is not sufficient or convincing.

In summary, LLMs in the SFLSCDP methodology provide clear benefits, and deploying the latest LLMs makes it realistic to expect even better results. The weak point, evidently, remains reproducibility. Therefore, appropriate protocols will have to be developed for LLM-based scientific research, but this topic exceeds the scope of this paper.

To conclude this section, the metrics issues (see research question III) need some final discussion. The presented DPEC and IPEC metrics provide a promising path, but it is evident that further refinement is needed. One option for crypto protocol design (perhaps a surprising one, but well aligned with the standard Alice and Bob notation) would be to use the size of the HLSPL file (specification of a protocol). Another option would be to use a standardized sandbox environment, deployable as a web service, to which such protocols are uploaded in Alice and Bob notation and evaluated accordingly.

Another lesson learned from the presented approach is that approximate power (and energy) consumption of crypto protocols is multidimensional, so some weighted combination of the mentioned approaches is likely to be the most suitable. However, this is a matter for further research and is also beyond the scope of this paper. 

Finally, considering the nature of these metrics and the evaluated protocols, it is reasonable to state that protocols whose GE is on the order of 10^5^ are considered lightweight.

## 6. Conclusions

The number of IoT devices is increasing significantly, and these devices will likely soon make up most of the Internet. At the same time, a large proportion of these devices have limited computing and energy resources. Moreover, many IoT devices operate in diverse environments, which makes them vulnerable to numerous kinds of attacks. Because they are often battery-powered, their frequent disposal also presents an environmental threat with sustainability implications. In addition, the advent of quantum computing jeopardizes the cryptographic primitives most used in current networks, which is also linked to broader issues surrounding sustainable computing solutions.

This paper addresses the above problems by presenting a methodological basis for the secure and sustainable development of cryptographic protocols, called the Sustainability-focused LLM-supported crypto protocols development process (SFLSCDP). This procedural approach is accompanied by the necessary metrics for evaluating lightweight solutions, i.e., direct power/energy consumption (DPEC) and indirect power/energy consumption (IPEC). DPEC is expressed in logic gate equivalents, while IPEC is expressed in the running times of cryptographic primitives. Although these metrics are approximate, they still require additional research to further improve quantitative evaluations of cryptographic protocols and the resources they require during their design. Some possible paths for the necessary research are discussed in the paper.

Furthermore, SFLSCDP integrates LLMs into the derivation and optimization of crypto-protocol design, alongside traditional formal verification techniques. In doing so, it appropriately addresses lightweight and sustainability issues, with LLMs contributing foresight capabilities that are central to such efforts.

Finally, using the described approach, the article presents a novel ultra-lightweight authenticated key exchange protocol, called UL-AKE, which has two variants: a lighter one (computationally less demanding but less secure) and a second one that provides better security but is computationally more demanding. Interestingly, by applying SFLSCDP, another variant was found, called UL-AKE key derivation, which is also lightweight, secure, and aligned with modern principles of AKE security protocol design.

The UL-AKE family is based on strong one-way hash functions, which makes it resistant to quantum computing, and has been formally verified with AVISPA. Its performance is evaluated using the presented metrics, where the UL-AKE family compares very well with its competitors and, in particular, its variant 1 qualifies as ultra-lightweight (a sensible definition of when a protocol can be classified as ultra-lightweight is also given). This family also represents a further contribution to the growing arsenal of methods and cryptographic solutions tailored to the IoT, and helps to satisfy the constant need for an ever-expanding arsenal of sustainable computing solutions.

## Figures and Tables

**Table 1:** The performance comparison of the protocols analyzed (GE for VII includes only phase 1, for VIII it includes all communications and tag cost, while for UL-AKE its complete cost is given).

I	II	III	IV	V	VI
5 *T_H_*, 4 *T_XOR_*	5 *T_H_*, 4 *T_XOR_*	15 *T_H_*, 5 *T_XOR_*	5 *T_H_*, 5 *T_XOR_*	10 *T_H_*, 4 *T_XOR_*	public-key ops
VII	VIII		UL-AKE v 1 (entity A left/entity B right)		
5 *T_H_*, 3 *T_XOR_*	4 *T_H_*, 5 *T_XOR_*		2 *T_H_*, 10 *T_XOR_*		1 *T_H_*, 4 *T_XOR_*
459,108 GE	107,772 GE		110,480 GE		

## Data Availability

All data are given or referenced in the article.

## Appendix A. SFLSCDP Application Details

The necessary step in our proactive threat model process was performed offline, without using an online large language model (LLM) such as ChatGPT, because of privacy concerns (our solution could be exposed to the public before its publication).

We deployed a local LLM, ggml-org/gemma-4-31B-it-GGUF:Q4_K_M, obtained via Hugging Face, on an HP Z2 Mini G1 with a Ryzen AI MAX+ 395, 128 GB RAM, and 8 TB VRAM. This LLM, created by Google DeepMind, is multimodal with a context window of up to 256 tokens. The Gemma family of models exhibits notable reasoning capabilities (in addition to supporting agentic and coding applications) while remaining deployable on local computers. The model has a total of 30.7B parameters, 60 layers, a sliding window of 1K tokens, and a context length of 256 tokens. The vocabulary size is 262K.

The prompts for in-context learning of the mentioned model were as follows:

I. The UL-AKE variant 1 protocol description follows: (the description from Section 3.1 is given here).
II. Your understanding of the protocol is OK. Proceed with security analysis of this UL-AKE and find possible threats within the Dolev–Yao model (if there are any). Put another way, state attacks that would compromise the protocol (if there are any).
III. Modify message 3 so that a long-term secret leak is prevented.
IV. Specify in CAS notation the fully corrected protocol (AVISPA-Safe Version). Make sure it is correct.
V. Now state those attacks that would compromise the protocol, but which fall out of the scope of the Dolev–Yao model.
VI. Consider now all possible disruptive technologies and developments that could affect Internet of Things devices’ security. State these disruptive technologies and developments.
VII. Do any of the just-found threats fall within the scope of the Dolev–Yao model? Put another way—would AVISPA classify UL-AKE as safe in the light of these new threats, so they would be undetected?
